# The prognostic role of tissue TLR2 and TLR4 in colorectal cancer

**DOI:** 10.1007/s00428-020-02833-5

**Published:** 2020-05-19

**Authors:** Ines Beilmann-Lehtonen, Camilla Böckelman, Harri Mustonen, Selja Koskensalo, Jaana Hagström, Caj Haglund

**Affiliations:** 1 Department of Surgery, University of Helsinki and Helsinki University Hospital HUS, Haartmaninkatu 4, PO Box 440, FIN-00029 Helsinki, Finland; 2 Translational Cancer Medicine Research Program, Faculty of Medicine, University of Helsinki, Helsinki, Finland; 3 Department of Pathology, University of Helsinki and Helsinki University Hospital, Helsinki, Finland

**Keywords:** Toll-like receptor 2, Toll-like receptor 4, Colorectal cancer, Colon cancer, Immunohistochemistry

## Abstract

Colorectal cancer (CRC), the second most common cancer globally, resulted in 881,000 deaths in 2018. Toll-like receptors (TLRs) are crucial to detecting pathogen invasion and inducing the host’s immune response. This study aimed to explore the prognostic value of TLR2 and TLR4 tumor expressions in colorectal cancer patients. We studied the immunohistochemical expressions of TLR2 and TLR4 using tissue microarray specimens from 825 patients undergoing surgery in the Department of Surgery, Helsinki University Hospital, between 1982 and 2002. We assessed the relationships between TLR2 and TLR4 expressions and clinicopathological variables and patient survival. We generated survival curves using the Kaplan-Meier method, determining significance with the log-rank test. Among patients with lymph node–positive disease and no distant metastases (Dukes C), a strong TLR2 immunoactivity associated with a better prognosis (*p* < 0.001). Among patients with local Dukes B disease, a strong TLR4 immunoactivity associated with a worse disease-specific survival (DSS; *p* = 0.017). In the multivariate survival analysis, moderate TLR4 immunoactivity compared with strong TLR4 immunoactivity (hazard ratio (HR) 0.66, 95% confidence interval (CI) 0.49–0.89, *p* = 0.007) served as an independent prognostic factor. In the multivariate analysis for the Dukes subgroups, moderate TLR2 immunoactivity (HR 2.63, 95% CI 1.56–4.44, *p* < 0.001) compared with strong TLR2 immunoactivity served as an independent negative prognostic factor in the Dukes C subgroup. TLR2 and TLR4 might be new prognostic factors to indicate which CRC patients require adjuvant therapy and which could spare from an unnecessary follow-up, but further investigations are needed.

**Electronic supplementary material:**

The online version of this article (10.1007/s00428-020-02833-5) contains supplementary material, which is available to authorized users.

## Introduction

Colorectal cancer (CRC) represents a major burden for public healthcare systems as the third most common malignancy globally and the second leading cause of cancer deaths. In 2018, more than 1.8 million new cases were diagnosed and 881,000 CRC deaths occurred [1]. A wide geographical difference in CRC burden exists, with a threefold higher incidence and about 55% of all deaths occurring in transitioning countries [1–3]. The CRC burden is expected to increase given the aging and growth of populations and the adoption of a so-called Western lifestyle [3].

CRC survival in developed countries has increased given improved screening and treatment, although 17% of stage II and 36% of stage III tumors still recur within 5 years. Identifying patients that need thorough follow-up and adjuvant therapy remains important, whereas other patients could be spared unnecessary treatment and possibly follow-up [4].

Chronic inflammation serves as an important risk factor for cancer. Both the local tumor microenvironment (TME) and the systemic host response play crucial roles in the development and progression of cancer [5, 6]. Reports have indicated that the host’s systemic inflammatory response (SIR), evidenced by an elevated preoperative C-reactive protein (CRP) value, predicts a shorter survival period among patients undergoing primary tumor resection [7, 8]. Yet, local inflammation found specifically in the invasive area of the tumor predicts better survival [9].

Toll-like receptors (TLR) represent a large family of pattern recognition receptors expressed on immune cells and epithelial cells and play a crucial role in the innate immune response since they detect pathogens and induce pro-inflammatory immune responses [5, 10–12]. Yet, TLR expression patterns vary across different immune cells. In endothelial cells, for example, TLR2 and TLR4 express intracellularly, whereas, in leukocytes such as monocytes, macrophages, neutrophils, dendritic cells (DCs), and natural killer (NK) cells, TLR2 and TLR4 expressed on the cell membranes [13].

TLRs work through the myeloid differentiation primary response gene 88 (myD88) pathway common to all TLRs. Furthermore, TLR3 and TLR4 work through the myD88-independent pathways [5, 10–12]. Additionally, TLRs induce the maturation of DCs leading to T cell activation and differentiation into effector cells, necessary for initiating an adaptive immune response. Thus, TLR signaling represents an important link between the host’s innate and adaptive immune responses [10, 14, 15].

Alongside exogenous microbial ligands, TLRs also recognize several endogenous host-derived ligands released during tissue damage and tumor progression [5, 10–13]. TLRs are also known to take part in the pathogenesis of several autoimmune, chronic inflammatory, and infectious diseases including rheumatoid arthritis [16], type 1 and 2 diabetes [17, 18], psoriasis [10], Crohn’s disease [19], and ulcerous colitis [20].

Several studies showed that TLRs play a role in cancer pathogenesis. TLRs express on tumor cells, while dying tumor cells release endogenous TLR ligands, thus activating TLR signaling and promoting tumorigenesis [21–23]. It is thought that different TLRs carry specific and different protumoral and/or antitumoral roles in different tumors [12]. In relation to CRC, no prognostic value has been found for TLR2, while contradictory results have emerged for TLR4 [24–27]. Therefore, this study aimed to clarify the prognostic roles of TLR2 and TLR4 in colorectal cancer.

## Materials and methods

### Patients

Our cohort consisted of 825 patients undergoing a primary operation for CRC at the Department of Surgery, Helsinki University Hospital, between 1982 and 2002. Among these patients, 457 (55.4%) were male. The median age was 67.5 years (interquartile range (IQR) 57.6–75.3 years). The tumor was located in the rectum in 401 (48.6%) patients and in the colon in 424 (51.4%) patients.

The median follow-up time was 5.1 years (IQR 1.2–17.2) and 641 (77.7%) patients died by the end of the follow-up period. The 5-year disease-specific survival (DSS) was 58.9% (95% confidence interval (CI) 55.4–62.4). Among right-sided cancer patients, 5-year DSS rates were 58.1% (95% CI 51.4–64.8) and 59.2% (95% CI 55.1–63.3) among left-sided cancer patients. Among 292 stage 2 patients, 25 (8.6%) received adjuvant therapy, while 53 (23.6%) of 225 stage 3 patients received adjuvant therapy, and 62 of 186 (33.3%) stage 4 patients received adjuvant therapy.

At the time of patient recruitment, we used the modified Dukes staging for CRC in our clinic. As such, 122 cases tumor were classified as Dukes stage A (14.8%), 292 cases as Dukes B (35.4%), 225 as Dukes C (27.3%), and 186 as Dukes D (22.5%). Table 1 summarizes the patients’ clinicopathological characteristics.

**Table 1:** Characteristics of colorectal cancer patients

Patient characteristics	*n* (%)
Age	
Median (IQR), years	67.5 (57.6–75.3)
Gender	
Male	457 (55.4)
Female	368 (44.6)
Dukes stage	
A	122 (14.8)
B	292 (35.4)
C	225 (27.3)
D	186 (22.5)
Tumor grade (WHO)	
1	26 (3.2)
2	563 (68.4)
3	201 (24.4)
4	33 (4.0)
Location	
Colon	424 (51.4)
Rectum	401 (48.6)
Side	
Right	225 (27.3)
Left	600 (72.7)
Histological type	
Adeno	734 (89.1)
Mucinous	90 (10.9)

*IQR*, interquartile range

We received the clinical data from patients’ medical records and the survival data and causes of death from the Population Register Center of Finland and Statistics Finland. The Surgical Ethics Committee of Helsinki University Hospital (Dnro HUS 226/E6/06, extension TMK02 §66 17.4.2013) approved the study protocol. The National Supervisory Authority of Health and Welfare granted permission to use tissue archive samples retrospectively without requiring individual consent (Valvira Dnro 10041/06.01.03.01/2012).

### Tissue samples

Surgical tumor samples fixed in formalin and embedded in paraffin were stored in the archives of the Department of Pathology at the University of Helsinki. Histopathologically representative areas of the tumor samples were marked on hematoxylin- and eosin-stained slides by an experienced pathologist (JH). From the annotated areas, three 1.0-mm cores were punched from each tumor and embedded in a recipient paraffin block with a semiautomatic tissue arrayer (Beecher Instruments Inc., Silver Spring, MD, USA). Sections of 4 μm were cut from the tissue microarray (TMA) blocks for immunohistochemistry.

### Immunohistochemistry

Slides with TMA block sections were deparaffinized in xylene and then rehydrated in solutions containing a decreasing concentration of ethanol, beginning with pure alcohol and ending with distilled water. The antigen retrieval was accomplished by treating the slides in a PreTreatment module (Lab Vision UK Ltd., UK) in a Tris-HCl buffer (pH 8.5) for 20 min at 98 °C.

Staining of the slides was carried out in an Autostainer 480 (Lab Vision, Fremont, CA, USA) using the REAL EnVision Detection System (peroxidase/DAB+, rabbit/mouse; Dako, Glostrup, Denmark). The inactivation of endogenous peroxidases was completed by incubating the slides in 0.3% hydrogen peroxide for 5 min. Subsequently, the primary incubation with rabbit polyclonal antibodies against TLR4 (H-80; Santa Cruz Biotechnology, Santa Cruz, CA, USA; diluted to 1:50) or TLR2 (H-175; Santa Cruz Biotechnology, Santa Cruz, CA, USA; diluted to 1:50) was carried out for 1 h, followed by incubation with the Dako REAL EnVision/HRP detection system using the Rabbit (ENV) reagent for 30 min. Finally, stainings were visualized using the Dako REAL DAB+ Chromogen for 10 min. Between each step, the slides were washed in PBS 0.04% Tween20. Slides were counterstained with Meyer’s hematoxylin and finally mounted in Pertex Mounting (Histolab Products AB, Sweden). Tissues showing a high immunoreactivity for these antigens were used as the positive controls (tonsillar and gums). Specimens processed without any primary antibody were used as the negative controls. We have compared the results of TLR4 stainings of a small cohort with the new TLR4 mouse monoclonal antibody (sc-293072, Santa Cruz Biotechnology, Santa Cruz, CA, USA), the immunoexpression of TLR4 by the two different antibodies correlated (*r*_s_ = 0.721, *p* < 0.001, Spearman’s rank correlation test).

### Scoring

**Fig. 1:**
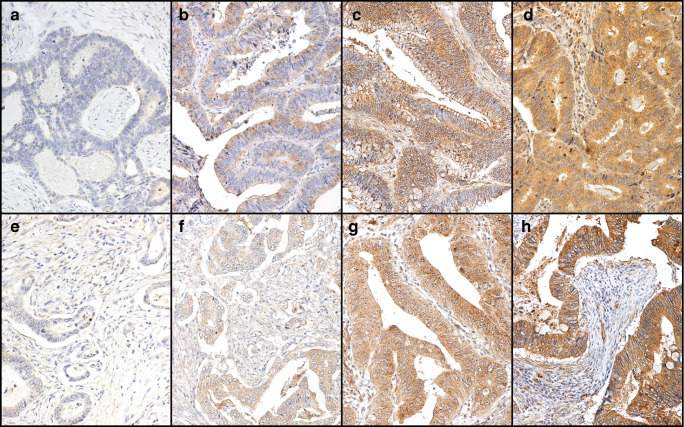
Images of TLR2 and TLR4 immunohistochemistry stainings representing colorectal cancer tumors with negative (**a**, **e**), weak (**b**, **f**), moderate (**c**, **g**), and strong (**d**, **h**) staining. Images **a**–**d** are stained with a TLR2 polyclonal antibody and images **e**–**h** are stained with a TLR4 polyclonal antibody. Original magnification × 20

Two researchers (IB-L and JH) independently scored the TLR2 and TLR4 immunostainings. TLR2 and TLR4 immunopositivity was defined as a brown cytoplasmic color in the tumor cells scored from 0 to 3 reflecting the staining intensity. Here, 0 represented negative staining and no immunoactivity, 1 represented a weak positive immunoactivity, 2 represented a moderate intensity of staining, and 3 represented a strong intensity of staining (Fig. 1). If three scores of the same patient were different, the highest score was used. The scores of both researchers were compared, and cases with any variance were assigned a final score reached through consensus.

### Statistical analysis

For the statistical analysis, we grouped TLR2 and TLR4 immunoexpressions into low (0), moderate (1–2), and high (3) expression levels. The associations between the TLR2 and TLR4 scoring and clinicopathological variables were analyzed used the chi-square test. DSS was calculated from the day of surgery to death from CRC. Survival curves were calculated using the Kaplan-Meyer method and the *p* values were calculated using the log-rank test.

For the univariate and multivariate survival analyses, we used the Cox proportional hazards model, adjusting the multivariate analysis for the tumor stage, differentiation, and location. The Cox model assumption of constant hazard ratios over time was tested through the inclusion of a time-dependent covariate separately for each variable tested.

A time-dependent variable was included for stage D, location, and differentiation to fulfill the Cox assumption. We also considered interaction terms, finding no significant interactions in the TLR4 model, although the TLR2 model identified a significant interaction between stage C and TLR2 immunoexpression. Thus, we performed a separate analysis for stage C patients.

We considered *p* < 0.05 as statistically significant and applied two-tailed tests. All statistical analyses were performed using SPSS version 24.0 (IBM SPSS Statistics, version 24.0 for Mac; SPSS, Inc., Chicago, IL, USA).

## Results

### Immunostaining for TLR2

Among 825 CRC tumor samples, interpreting the TLR2 immunostaining proved successful in 775 (93.9%) samples. The immunostaining of TLR2 was not possible to interpret appropriately in 6.1% of patients due to technical staining problems or due to missing cancer tissue.

TLR2 stained evenly in the cytoplasm. The immunopositivity was scored as strong in 200 (25.8%) samples, moderate in 313 (40.4%), and weak in 209 (27.0%), and no immunopositivity was identified in 53 (6.8%) samples. In the statistical analysis, patients fell into three groups, scored as 0 (negative), 1–2 (mild and moderate), and 3 (high; Fig. 1a–d).

### Immunostaining for TLR4

TLR4 stained evenly in the cytoplasm of 769 (93.2%) samples. The immunostaining of TLR4 was not possible to interpret in 6.8% of patients for reasons mentioned above for TLR2. The immunopositivity was scored as strong in 101 (13.1%) samples, moderate in 358 (46.6%), weak in 273 (35.5%), and no immunopositivity was identified in 37 (4.8%) samples. Here, patients fell into three groups, scored as 0 (negative), 1–2 (mild and moderate), and 3 (high; Fig. 1e–h).

### Association with clinicopathological parameters

Table 2 summarizes the relationships between the clinicopathological variables and TLR2 immunointensity, while Table 3 provides the relationships with the TLR4 immunointensity. TLR2 associated with the tumor location (*p* = 0.042) and the tumor grade (*p* = 0.004, chi-square test, Table 2). TLR4 associated with gender (*p* = 0.006) and the tumor grade (*p* < 0.001, chi-square test, Table 3). Neither TLR2 nor TLR4 immunoexpression associated with patient age, the histological type of tumor, or the Dukes tumor stage.

**Table 2:** Association between TLR2 immunointensity and clinicopathological parameters among colorectal cancer patients

Clinicopathological variable	*n*	TLR			*p* value^1^
		0	1 and 2	3	
		*n* (%)	*n* (%)	*n* (%)	
Age					
< 65	330	23 (7.0)	217 (65.8)	90 (27.3)	0.701
≥ 65	445	30 (6.7)	305 (68.5)	110 (24.7)	
Gender					
Male	428	29 (6.8)	296 (69.2)	103 (24.1)	0.452
Female	347	24 (6.9)	226 (65.1)	97 (28.0)	
Side					
Right	217	19 (8.8)	138 (63.6)	60 (27.6)	0.266
Left	558	34 (6.1)	384 (68.8)	140 (25.1)	
Location					
Colon	403	28 (6.9)	256 (63.5)	119 (29.5)	0.042
Rectum	372	25 (6.7)	266 (71.5)	81 (21.8)	
Histological type					
Adeno	689	42 (6.1)	467 (67.8)	180 (26.1)	0.058
Mucinous	85	11 (12.9)	55 (64.7)	19 (22.4)	
Dukes stage					
A	110	12 (10.9)	76 (69.1)	22 (20.0)	0.537
B	279	16 (5.7)	189 (67.7)	74 (26.5)	
C	210	14 (6.7)	139 (66.2)	57 (27.1)	
D	176	11 (6.3)	118 (67.0)	47 (26.7)	
Tumor grade (WHO)					
1	24	5 (20.8)	11 (45.8)	8 (33.3)	0.004
2	529	33 (6.2)	356 (67.3)	140 (26.5)	
3	189	9 (4.8)	135 (71.4)	45 (23.8)	
4	31	6 (19.4)	18 (58.1)	7 (22.6)	

*TLR2*, Toll-like receptor 2

^1^Chi-square test

**Table 3:** Association between TLR4 immunointensity and clinicopathological parameters in colorectal cancer patient

Clinicopathological variable	*n*	TLR4			*p* value^1^
		0	1 and 2	3	
		*n* (%)	*n* (%)	*n* (%)	
Age					
< 65	323	20 (6.2)	264 (81.7)	39 (12.1)	0.204
≥ 65	443	16 (3.6)	365 (82.4)	62 (14.0)	
Gender					
Male	428	28 (6.5)	354 (82.7)	46 (10.7)	0.006
Female	341	9 (2.6)	277 (81.2)	55 (16.1)	
Side					
Right	214	13 (6.1)	177 (82.7)	24 (11.2)	0.4
Left	555	24 (4.3)	454 (81.8)	77 (13.9)	
Location					
Colon	404	18 (4.59)	330 (81.7)	56 (13.9)	0.748
Rectum	365	19 (5.2)	301 (82.5)	45 (12.3)	
Histological type					
Adeno	689	29 (4.2)	570 (82.7)	90 (13.1)	0.061
Mucinous	79	8 (10.1)	60 (75.9)	11 (13.9)	
Dukes stage					
A	114	9 (7.9)	90 (78.9)	15 (13.2)	0.342
B	273	8 (2.9)	223 (81.7)	42 (15.4)	
C	208	11 (5.3)	175 (84.1)	22 (12.6)	
D	174	9 (5.2)	143 (82.2)	22 (12.6)	
Tumor grade (WHO)					
1	25	1 (4.0)	22 (88.0)	2 (8.0)	< 0.001
2	528	23 (4.4)	431 (81.6)	74 (14.0)	
3	185	5 (2.7)	158 (85.9)	21 (11.4)	
4	30	8 (26.7)	19 (63.3)	3 (10.0)	

*TLR4*, Toll-like receptor 4

^1^Chi-square test

### Survival analysis

In the DSS analysis, we found no difference between the different TLR2 (*p* = 0.199, log-rank test) or TLR4 expression groups (*p* = 0.240, log-rank test; data not shown).

**Fig. 2:**
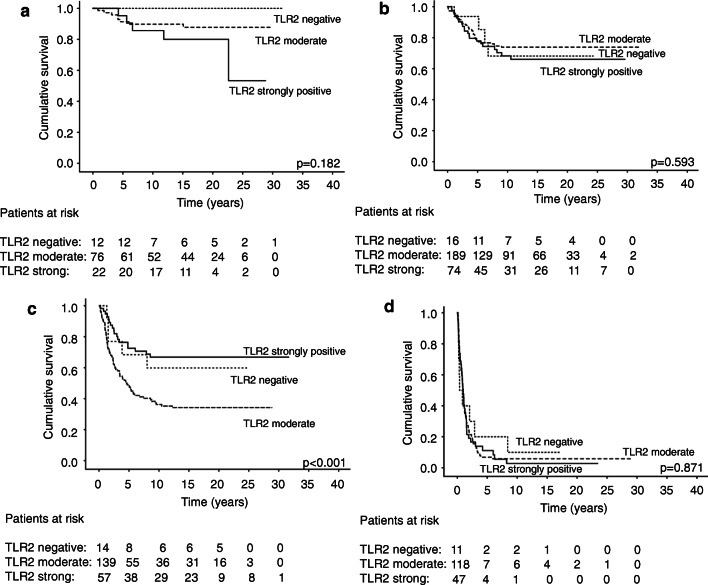
Disease-specific survival analysis of TLR2 in colorectal cancer patients using the Kaplan-Meier method. **a** Dukes A, **b** Dukes B, **c** Dukes C, **d** and Dukes D patients. The log-rank test was used

In the subgroup analysis, TLR2 emerged as a prognostic factor among the Dukes C patients, that is, those with lymph node–positive but distant metastasis–free disease (Fig. 2c; *p* < 0.001, log-rank test). In this subgroup, the 5-year DSS was 68.4% (95% CI 42.7–94.1) among patients with a negative TLR2 immunostaining and 72.6% (95% CI 60.6–84.6) among those with a strong TLR2 immunostaining compared with 48.2% (95% CI 39.2–57.3) among patients with a moderate TLR2 immunostaining. Among the subgroups, Dukes A (Fig. 2a), B (Fig. 2b), and D (Fig. 2d) patients, TLR2 did not serve as a prognostic factor.

**Fig. 3:**
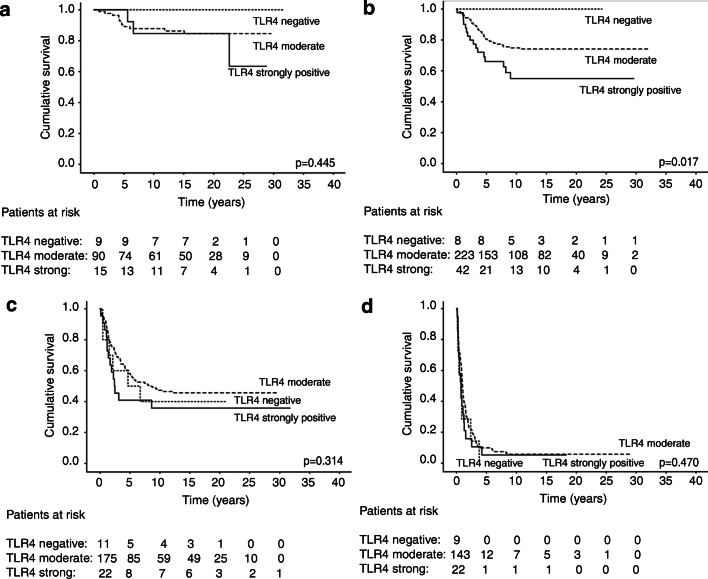
Disease-specific survival analysis of TLR4 in colorectal cancer patients using the Kaplan-Meier method. **a** Dukes A, **b** Dukes B, **c** Dukes C, **d** and Dukes D patients. The log-rank test was used

By contrast, TLR4 immunostaining served as a negative prognostic factor among Dukes B patients (Fig. 3b; *p* = 0.017, log-rank test). A 5-year DSS reached 100% among TLR4-negative patients, 80.6% (95% CI 75.1–86.1) among TLR4-moderate patients, and 66.1% (95% CI 51.0–81.2) among TLR4-strong immunostaining patients. Among the Dukes A (Fig. 3a), Dukes C (Fig. 3c), and Dukes D (Fig. 3d) subgroups, TLR4 did not serve as a prognostic factor.

In the subgroup analysis among patients who did not receive adjuvant therapy, the results mirrored those among the entire cohort. Among Dukes C patients who did not receive adjuvant therapy, a very strong TLR2 expression predicted a better survival compared with those patients with a moderate TLR2 expression (Suppl. Fig. 2c; *p* < 0.001, log-rank test). Among Dukes B patients who did not receive adjuvant therapy, a strong TLR4 immunoexpression showed a worse prognosis (Suppl. Fig. 3b; *p* = 0.01, log-rank test). In the Cox univariate survival analysis, patients over 65 years of age (*p* = 0.001), patients with less differentiated cancer (*p* = 0.001), and patients with higher Dukes stages (*p* < 0.001) exhibited worse prognoses. In the Cox multivariate survival analysis adjusted for age, gender, Dukes stage, the tumor grade, and tumor location, a moderate TLR4 immunoreactivity (HR 0.66, 95% CI 0.49–0.89, *p* = 0.007) compared with a strong TLR4 tumor expression served as an independent prognostic factor (Table 4). In the Cox multivariate analysis including the same clinicopathological parameters and TLR2, the TLR2 immunoreactivity did not serve as prognostic factor (data not shown). In the Cox multivariate analysis for the Dukes C subgroup, a moderate TLR2 immunoactivity (HR 2.63, 95% CI 1.56–4.44, *p* < 0.001) compared with a strong TLR2 immunoreactivity served as an independent negative prognostic factor (Table 5). In the Cox multivariate analysis, adjuvant therapy did not serve as prognostic factor (Suppl. Table 1, *p* = 0.747).

**Table 4:** Univariate and multivariate Cox regression analysis for disease-specific survival of colorectal cancer patients

	Univariate analysis		*p* value^1^	Multivariate analysis		*p* value^1^
	Hazard ratio	95% CI		Hazard ratio	95% CI	
Age						
< 65	1.00			1.00		
≥ 65	1.42	1.14–1.75	0.001	1.91	1.52–2.40	< 0.001
Gender						
Male	1.00			1.00		
Female	0.96	0.78–1.18	0.71	1.4	0.91–1.42	0.248
Dukes stage						
A	1.00			1.00		
B	2.13	1.35–4.2	0.005	2.33	1.32–4.09	0.003
C	5.3	3.74–11.2	< 0.001	6.46	3.74–11.17	< 0.001
D	38.24	23.9–83.1	< 0.001	44.62	23.9–83.3	< 0.001
Tumor grade (WHO)						
1–2	1.00			1.00		
3–4	2.49	1.81–3.43	0.001	2.13	1.52–3.0	< 0.001
Location						
Colon	1.00			1.00		
Rectum	1.16	1.06–1.27	0.001	0.87	0.64–1.2	0.416
TLR4						
Strong	1.00			1.00		
Moderate	0.8	0.59–1.07	0.127	0.66	0.49–0.89	0.007
Negative	0.67	0.37–1.24	0.202	0.67	0.36–1.25	0.206

Multivariate analysis included adjustments for gender, age, Dukes stage, and tumor grade

*TLR4*, Toll-like receptor 4; *CI*, confidence interval

**Table 5:** Multivariate Cox regression analysis for disease-specific survival of Dukes C subgroup colorectal cancer patients

TLR2 expression	Hazard ratio	95% CI	*p* value^1^
Strong	1.00		
Moderate	2.63	1.56–4.44	< 0.001
Negative	1.26	0.47–3.4	0.646

Multivariate analysis included adjustment for gender, age, Dukes stage, and tumor grade

*TLR2*, Toll-like receptor 2; *CI*, confidence interval

## Discussion

Among CRC patients with lymph node metastases but no distant metastases (Dukes C), a very strong expression of TLR2 predicted a remarkably better survival compared with those patients with a moderate expression. Among patients with a Dukes B tumor, a strong TLR4 immunoexpression indicated a worse prognosis. A survival analysis among patients who did not receive adjuvant therapy revealed similar results. A strong TLR4 expression in the tumor served as an independent prognostic factor.

CRC patients with tumors that have progressed to regional lymph nodes or metastasized to other organs are typically treated with postoperative chemotherapy. However, a portion of such patients still die from recurrent disease. Yet, some patients with lymph node–positive disease survive cancer without adjuvant treatment and some patients with seemingly local disease die from recurrent disease. Thus, discussions in the literature continue regarding which patients should receive adjuvant therapy and which may be spared therapy [4, 28]. Here, we have identified a potential role for prognostic biomarkers such as TLRs.

A recent study investigated the roles of TLR2 and TLR4 in 118 CRC patients [24]. Among such patients, the intensity and extent of staining were studied separately in the tumor, the invasive border, the normal mucosa, and lymph node metastases if present. TLR4 expression was significantly stronger in the normal mucosa and in lymph node metastases compared with that in the tumor. The opposite emerged for TLR2 expression, which increased in carcinoma cells and stained at a weaker intensity in the lymph nodes than in the bulk and invasive front of the tumor. In that study in contrast to our findings here, researchers found no significant association between TLR2 and TLR4 tumor expressions and patient survival. In their study, TLR2 and TLR4 were expressed in all tumors, whereas in our series, a few tumors lacked TLR expression.

In another study, Nihon-Yanagi et al. found a higher TLR2 expression in cancer samples at each stage compared with that in the normal mucosa [26]. Furthermore, TLR2 was more intensively expressed in stage II and III tumors. In their study, TLR4 expression in carcinoma tissues mirrored that in the normal mucosa. The discrepancy between these findings and our results may be explained by their smaller patient sample size (*n* = 50), the different detection methods, and the different antibodies used. For instance, Nihon-Yanagi et al. studied the TLR4 and TLR2 expressions using real-time PCR and TLR immunoexpression from whole sections, whereas we used TMA slides and immunohistochemistry alone.

Additionally, Eiró et al. found that a high TLR4 expression in CRC tumor cells relates to a lower recurrence rate [29]. Our findings did not agree with this result. While we used the same antibody, we found that a strong TLR4 expression predicted a worse prognosis in lymph node–negative disease. Eiró et al. reported 63 Dukes B diseased patients from a patient cohort of 104. Additionally, they report of 70 lymph node–positive patients from the same cohort of 104, a figure which appears somewhat problematic. In their study, TLR4 positivity was located on the cell membranes, whereas we found cytoplasmic expression.

In another study, Siminatonaki et al. using Western blot and immunohistochemistry found a positive correlation between TLR4 downregulation and the occurrence of lymphogenous and hematogenous metastases in 115 CRC patients [27]. In our study, however, among Dukes B patients, TLR4 overexpression indicated a worse prognosis, although among Dukes C and D tumors, we found no significant relationship. Siminatonaki et al. additionally reported a TLR4 positivity in both the cytoplasm and cell membranes, whereas we observed immunopositivity primarily in the cytoplasm, despite using the same antibody.

TLR signaling plays an important role in different cancers and the role of TLR expression can apparently vary between different cancers [12]. In Barrett’s metaplasia, the TLR4 protein expression appears upregulated, with the expression intensity correlating with a poor prognosis and with the degree of dysplasia in esophageal squamous cell carcinoma development [30]. During the early stage of oral tongue squamous cell carcinoma, TLR2 and TLR4 expressions serve as predictive markers of invasive tumor growth and a higher tumor grade [31]. The nuclear TLR2 expression level can predict tumor recurrence and neck metastases of tongue cancer [32]. Previous studies demonstrated that, in pancreatic ductal adenocarcinoma, TLR2 and TLR4 serve as positive predictors of prognosis in stage I and II disease and in patients with small-size tumors [33]. In murine and human gastric tumors, TLR2 appears upregulated and associates with a poorer overall survival in humans [34].

An increased TLR4 expression has been associated with tumor size and distant metastases in breast cancer [35], a higher recurrence rate in prostate cancer [36], and a poorer overall survival rate in ovarian cancer [37]. Additionally, an increased TLR4 expression associated with a shorter relapse-free survival time in cutaneous malignant melanomas [25], a poorer differentiation state in lung cancer [38], and a larger tumor size in murine experimental melanoma lung metastases [39]. Interestingly, in follicular thyroid neoplasms, both the downregulation and upregulation of TLR4 have been connected to primary metastases and the aggressiveness of cancer, whereas TLR2 expression associated with no clinicopathological parameter [40].

Based on our findings, a very strong TLR2 expression served as a positive prognostic factor among patients with lymph node–positive disease (Dukes C stage). Additionally, patients who had a negative TLR2 expression exhibited a better DSS than patients with moderate TLR staining, although the number of patients with a negative TLR2 expression in the Dukes C subgroup was too small to draw any definitive conclusions. To our knowledge, this favorable TLR2 prognostic role in CRC has not been previously reported. TLR2 signaling may generate both pro-inflammatory and anti-inflammatory responses. The interaction between TLR2 and different co-receptors such as TLR1, TLR6, CD36, CD14, and Dectin-1 renders the TLR2 response even more complicated [41].

We found that a strong TLR4 immunoreactivity predicts a worse prognosis in patients with a local colorectal tumor. More interestingly, among patients in the subgroups of Dukes A and B tumors (patients with no lymph node metastases), none of the patients lacking TLR4 expression in the tumor died from the disease. The number of patients in these subgroups was, however, quite small, such that further studies on larger cohorts are necessary.

Finding biomarkers remains pivotal in order to detect high-risk disease and to identify patients requiring adjuvant therapy. Our findings suggest that a strong TLR4 immunopositivity could identify those stage II patients requiring adjuvant therapy. A negative TLR4 immunostaining, however, could possibly spare some lower stage patients from unnecessary follow-up. In lymph node–positive disease, both a negative and a very strong TLR2 expression might serve as a predictive marker to identify patients that could be spared from adjuvant therapy.

The strength of our work lies in the large and well-characterized patient cohort, which allowed us to focus on different stage subgroups. The follow-up time in this cohort is also rather long, which allowed us to determine if patients were genuinely cured of cancer. On the other hand, the cohort is quite old. One strength to using an older cohort lies in the less frequent administration of adjuvant therapy at that time, whereby we can observe a more natural course of disease following surgery. Moreover, one drawback to using an older cohort lies in the harvesting of lymph nodes from surgical specimens, which relied on significantly inferior practices than those used today. In older series, thus, there might be a small migration of stage [42]. Unsurprisingly, surgical techniques have changed somewhat and operations along embryological planes, total mesorectal excision (TME), and complete mesocolic excision (CME) now represent standard procedures in CRC surgery [43, 44]. Another disadvantage to using an older cohort lies in the different staging classifications used at that time compared with the staging methods currently employed. The TMA technique allows the analysis of a large patient cohort, although inadequate representation of the whole tissue section might represent a limitation of this technique. By punching multiple areas of the primary tumor, however, we minimize this limitation [45].

## Conclusions

To our knowledge, this represents the first study to report a predictive role of TLR2 in CRC. A strong TLR2 immunoexpression served as a positive prognostic factor in lymph node–positive CRC patients. A strong TLR4 immunoexpression, however, predicted a worse prognosis in patients with local CRC. In particular, none of the patients with a local, TLR4-negative tumor died from the disease. Further investigations are necessary in order to validate these findings.

## Electronic supplementary material

ESM 1 (DOCX 19 kb)

Supplementary Fig. 1 Images of TLR2 negative staining (**a**), TLR4 negative staining (**b**), TLR2 positive control staining (**c**), TLR4 positive control staining (**d**) (PNG 2325 kb) (PNG 2325 kb)

High Resolution Image (EPS 8861 kb)

Supplementary Fig. 2 Disease-specific survival analysis of TLR2 in colorectal cancer patients not treated with adjuvant therapy using the Kaplan-Meier method. (**a**) Dukes A, (**b**) Dukes B, (**c**) Dukes C, (**d**) and Dukes D patients. The log-rank test was used (PNG 249 kb) (PNG 249 kb)

High Resolution Image (EPS 1443 kb)

Supplementary Fig. 3 Disease-specific survival analysis of TLR4 in colorectal cancer patients not treated with adjuvant therapy using the Kaplan-Meier method. (**a**) Dukes A, (**b**) Dukes B, (**c**) Dukes C, (**d**) and Dukes D patients. The log-rank test was used (PNG 229 kb)

High Resolution Image (EPS 1353 kb)

## Abbreviations

TLR: Toll-like receptor
TLR2: Toll-like receptor 2
TLR4: Toll-like receptor 4
CRC: Colorectal cancer
CI: Confidence interval
HR: Hazard ratio
IQR: Interquartile range
TMA: Tissue microarray
CRP: C-reactive protein
TME: Tumor microenvironment
SIR: Systemic inflammatory response
DSS: Disease-specific survival
DC: Dendritic cell
MyD88: Myeloid differentiation primary response gene 88
IBD: Inflammatory bowel disease
NF-휅B: Nuclear factor 휅B
