# Supplementary material for: The prognostic role of tissue TLR2 and TLR4 in colorectal cancer
Source: Virchows Arch. 2020 May 19;477(5):705–15. doi: 10.1007/s00428-020-02833-5 (PMC7581516; doi:10.1007/s00428-020-02833-5)
Supplement: Supplementary file 1 — (DOCX 19 kb) [file 428_2020_2833_MOESM1_ESM.docx]

|  | **Univariate analysis** | |  | | **Multivariate analysis** | |  |
| --- | --- | --- | --- | --- | --- | --- | --- |
|  | **Hazard ratio** | **95% CI** | ***p* value^1^** | | **Hazard ratio** | **95% CI** | ***p* value^1^** |
| Age |  |  |  | |  |  |  |
| <65 | 1.00 |  |  | | 1.00 |  |  |
| ≥65 | 1.42 | 1.14–1.75 | 0.001 | | 1.91 | 1.52–2.40 | <0.001 |
|  |  |  |  | |  |  |  |
| Gender |  |  |  | |  |  |  |
| Male | 1.00 |  |  | | 1.00 |  |  |
| Female | 0.96 | 0.78–1.18 | 0.710 | | 1.14 | 0.91–1.42 | 0.248 |
|  |  |  |  | |  |  |  |
| Dukes stage |  |  |  | |  |  |  |
| A | 1.00 |  |  | | 1.00 |  |  |
| B | 2.13 | 1.35–4.2 | 0.005 | | 2.33 | 1.32–4.09 | 0.003 |
| C | 5.3 | 3.74–11.2 | <0.001 | | 6.46 | 3.74–11.17 | <0.001 |
| D | 38.24 | 23.9–83.1 | <0.001 | | 44.62 | 23.91–83.27 | <0.001 |
|  |  |  |  | |  |  |  |
| Tumor grade (WHO) |  |  |  | |  |  |  |
| 1–2 | 1.00 |  |  | | 1.00 |  |  |
| 3–4 | 2.49 | 1.81–3.43 | 0.001 | | 2.13 | 1.52–2.00 | <0.001 |
|  |  |  |  | |  |  |  |
| Location |  |  |  | |  |  |  |
| Colon | 1.00 |  |  | | 1.00 |  |  |
| Rectum | 1.16 | 1.06–1.27 | 0.001 | | 1.15 | 1.05–1.27 | 0.396 |
| Adjuvant therapy |  |  |  | |  |  |  |
| Yes | 1.00 |  |  | | 1.00 |  |  |
| No | 1.35 | 0.98–1.87 | 0.065 | | 1.04 | 0.80–1.34 | 0.747 |
| TLR4 |  |  |  | |  |  |  |
| Strong | 1.00 |  |  | | 1.00 |  |  |
| Moderate | 0.8 | 0.59–1.07 | 0.127 | | 0.66 | 0.49–0.89 | 0.007 |
| Negative | 0.67 | 0.37–1.24 | 0.202 | | 0.67 | 0.36–1.25 | 0.206 |
|  |  |  |  | |  |  |  |
| Multivariate analysis adjusted for gender, age, Dukes stage, and tumor grade. | | | | | | |  |
| Abbreviations: TLR4, toll-like receptor 4; CI, confidence interval. | | | | | | | |
|  | | | |  |  |  |  |
|  | | | | | | |  |
|  | | | | | | |  |
|  |  |  |  | |  |  |  |
