# Supplementary figures and images for: The prognostic role of tissue TLR2 and TLR4 in colorectal cancer
Source: Virchows Arch. 2020 May 19;477(5):705–15. doi: 10.1007/s00428-020-02833-5 (PMC7581516; doi:10.1007/s00428-020-02833-5)

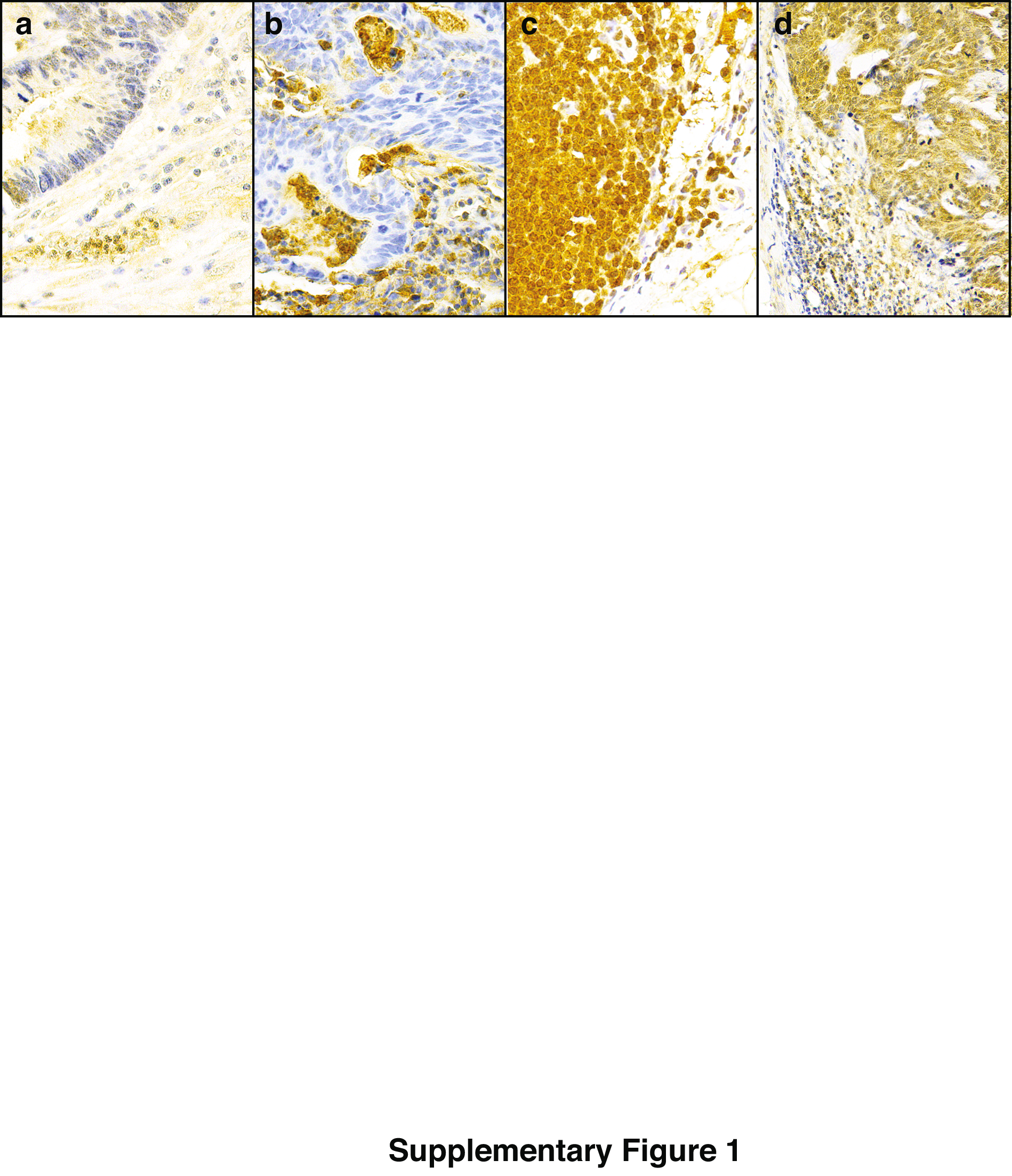

Supplement: Supplementary file 2 — Images of TLR2 negative staining (a), TLR4 negative staining (b), TLR2 positive control staining (c), TLR4 positive control staining (d) (PNG 2325 kb) (PNG 2325 kb) [file 428_2020_2833_Fig4_ESM.png]

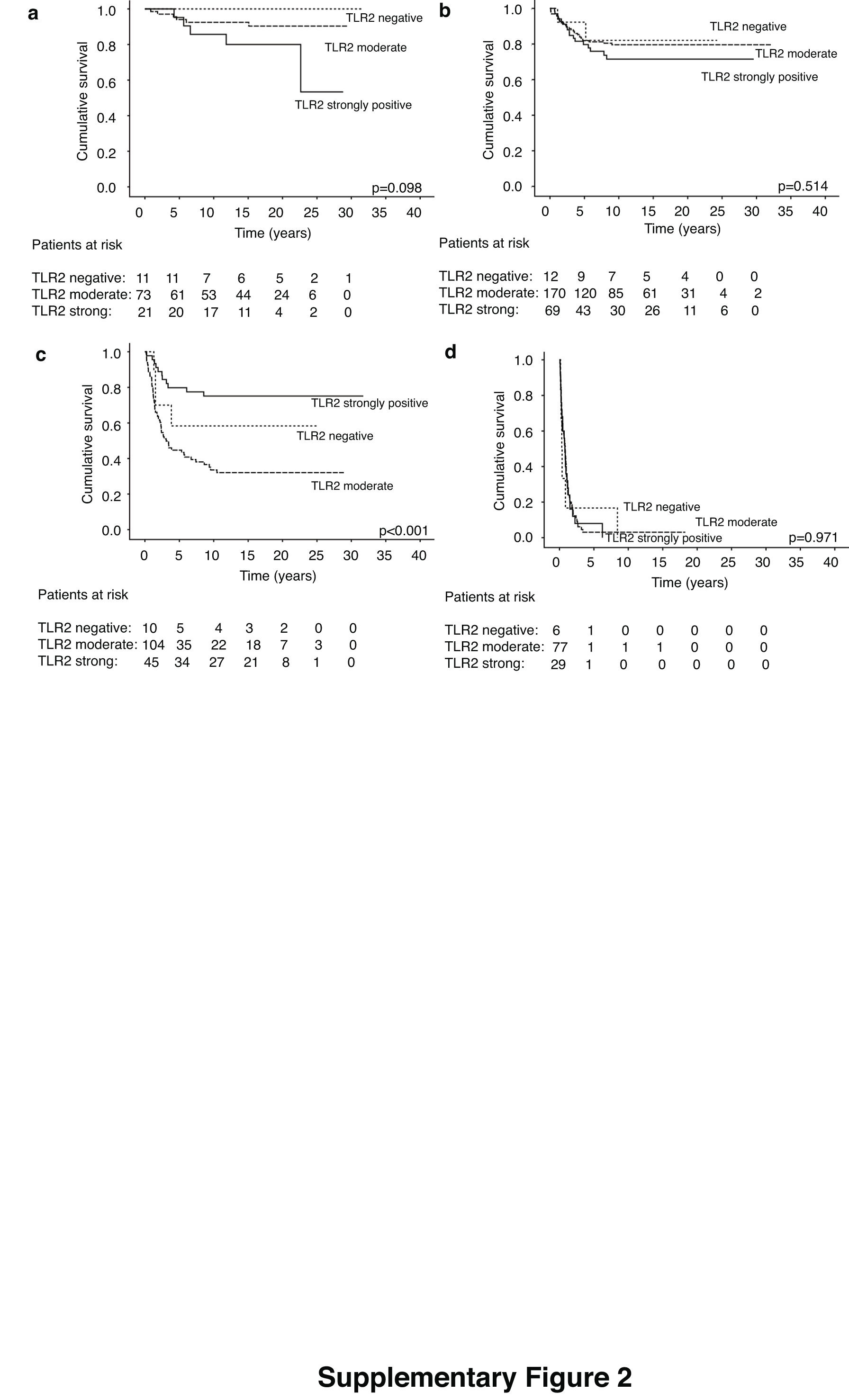

Supplement: Supplementary file 4 — Disease-specific survival analysis of TLR2 in colorectal cancer patients not treated with adjuvant therapy using the Kaplan-Meier method. (a) Dukes A, (b) Dukes B, (c) Dukes C, (d) and Dukes D patients. The log-rank test was used (PNG 249 kb) (PNG 249 kb) [file 428_2020_2833_Fig5_ESM.png]

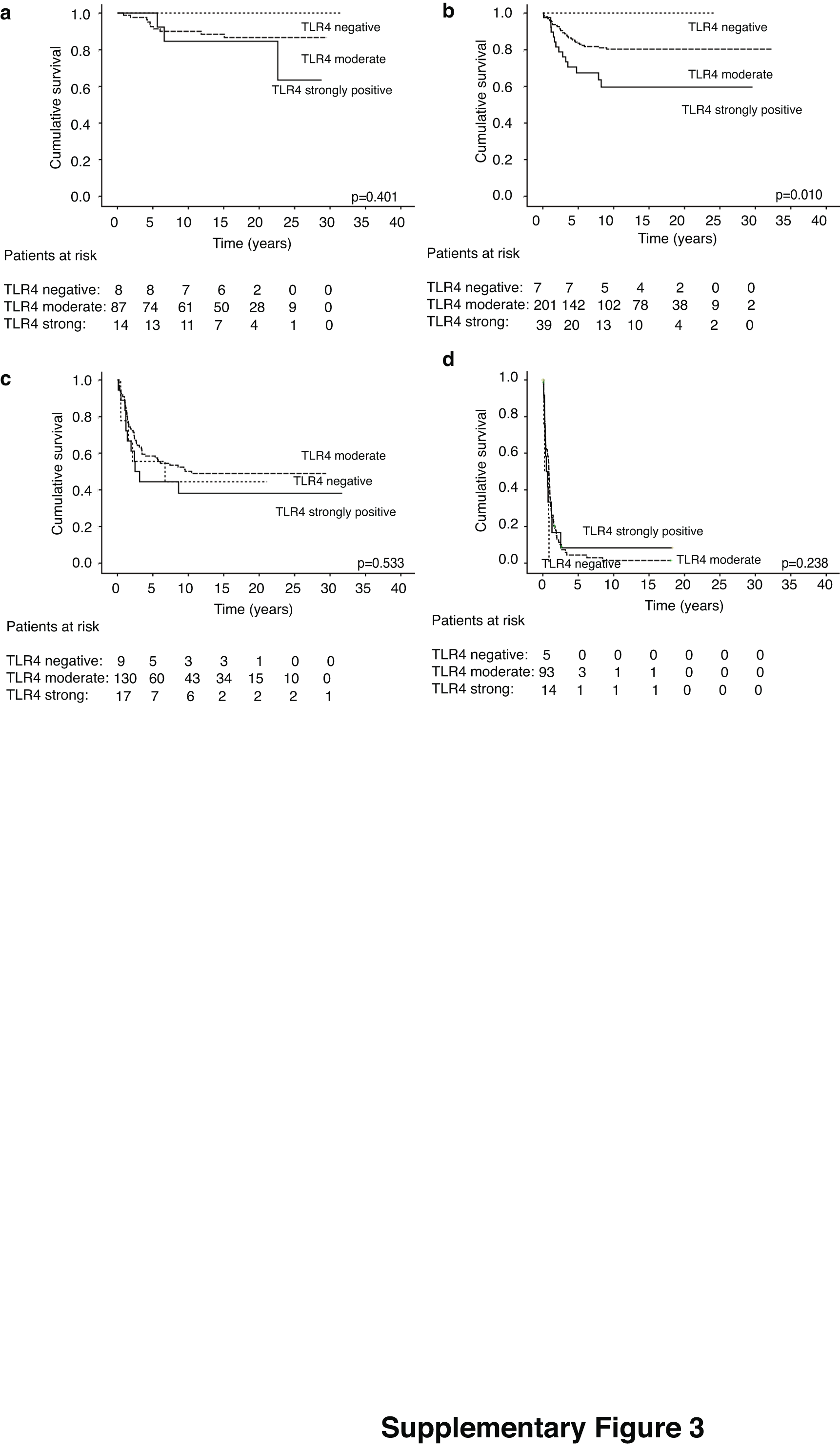

Supplement: Supplementary file 6 — Disease-specific survival analysis of TLR4 in colorectal cancer patients not treated with adjuvant therapy using the Kaplan-Meier method. (a) Dukes A, (b) Dukes B, (c) Dukes C, (d) and Dukes D patients. The log-rank test was used (PNG 229 kb) [file 428_2020_2833_Fig6_ESM.png]
